# Protocol for a review of statistical methods used to estimate risk ratios and risk differences in parallel cluster randomised trials

**DOI:** 10.1186/s13063-025-09395-4

**Published:** 2026-01-05

**Authors:** Jack A. Hall, Samuel I. Watson, Jon Bishop, Yixin Wang, Julia F. Shaw, Monica Taljaard, Karla Hemming

**Affiliations:** 1 Department of Applied Health Sciences, University of Birmingham, Birmingham, UK; 2 Birmingham Clinical Trials Unit, University of Birmingham, Birmingham, UK; 3 Methodological and Implementation Research Program, Ottawa Hospital Research Institute, Ottawa, ON Canada; 4 School of Epidemiology and Public Health, University of Ottawa, Ottawa, ON Canada

**Keywords:** Cluster randomised trial, Binary, Risk ratio, Risk difference, Review

## Abstract

**Background:**

Cluster randomised trials randomise groups of individuals, such as clinics, schools, or communities, and are used when interventions apply at the group level, when individual-level interventions risk contamination between participants, or to reflect real-world implementation. When outcomes are binary, treatment effects may be expressed as relative measures (such as odds ratios or risk ratios) or absolute measures (such as risk differences). CONSORT guidelines recommend reporting both, but risk ratios and risk differences are often underreported compared to odds ratios. Estimating these measures in cluster trials is more complex than in individually randomised trials, requiring appropriate handling of clustering, convergence issues, and small sample corrections. There is currently little empirical evidence describing which statistical methods are used to estimate these effect measures in published cluster trials.

**Methods:**

This protocol describes the planned methods for a methodological review of published cluster randomised trials. We will use an existing database of 800 trials conducted in low- and middle-income countries. From this, we will identify a subset of trials with a parallel design and a binary primary outcome. Trials reporting a risk ratio or risk difference for the primary outcome will undergo further detailed data extraction. We will summarise the methods used to estimate these effects, including how clustering and small sample sizes were handled, and whether estimates were adjusted for covariates.

**Discussion:**

This review will provide the first detailed description of how risk ratios and risk differences are currently estimated and reported in cluster randomised trials. The findings will inform the development of methodological guidance and help identify gaps in reporting and implementation. This is particularly important as interest grows in improving estimand specification and the clarity of statistical analysis plans.

## Background

Cluster randomised trials (CRTs) are a type of randomised study where groups, or clusters, of participants are randomised together. Cluster randomisation may be necessary if the experimental intervention is at the group level, such as a training or monitoring programme for clinical staff within healthcare facilities [1]; where there is interaction between individuals within a cluster, such as community-based interventions against communicable diseases [2]; or where it is difficult to ensure patients would receive their allocated treatment if they were randomised individually, such as changing a surgeon’s scrubbing strategy for reducing surgical site infections [3]. Cluster randomisation poses unique statistical challenges as outcomes are correlated within clusters. This occurs for two reasons. First, clusters are often defined using natural groups or boundaries, such as schools, GP practices or geopolitical regions, so the characteristics of patients within these groups are likely to be more similar than those from other groups. The second reason is that the clusters themselves impact the outcome, perhaps due to varying care processes or the quality of hospital personnel or equipment. As outcomes are likely to be correlated within a cluster and there is a correlation between treatment assignment within clusters (the entire cluster is assigned the same treatment allocation).the clustering should not be ignored in an analysis, as doing so would lead to inflated type 1 errors and invalid estimates of precision [4].

### Binary outcomes and choice of effect estimate

Binary outcomes account for over half of primary outcomes reported in randomised controlled trials (RCTs) and CRTs, and most trials report at least one secondary binary outcome [5, 6]. The impact of a treatment on a binary outcome may be summarised in relative (odds ratio (OR), risk ratio (RR)) or absolute terms (risk difference (RD), number needed to treat (NNT)), each with differing interpretations, benefits, and limitations [7]. The choice of measure can affect the perceived benefit of the therapeutic intervention, with patients and clinicians often interpreting treatment benefits differently when presented with absolute measures instead of relative ones [8–15], and trialists should ensure they appropriately represent the clinical significance, or lack thereof, of their results. Reporting an absolute measure is particularly important when outcomes are rare, as relative measures can exaggerate the perceived magnitude of treatment benefit [16], and presenting both is recommended by the CONSORT guidelines for RCTs and CRTs [17–20]. Despite this, since the publication of CONSORT, reviews of published reports for RCTs and CRTs have found that only 8%−20% of trials follow this guidance, and absolute effect measures are much less frequently reported than relative measures for binary outcomes [16, 21–24].

### Covariate adjustment

CRTs are more susceptible to internal bias than traditional RCTs due to the possibility of post-randomisation recruitment and the difficulty of effective blinding. In addition, the relatively low number of randomisation units increases the risk of chance imbalance in individual- and cluster-level covariates, which can complicate interpretation of results [25, 26]. The validity of effect estimates can be improved by adjusting for covariates that are associated with these biases, or where there appears to be a baseline imbalance [27, 28]. Adjusting for covariates used in restricted randomisation or other prognostic covariates may also improve precision [29, 30]. Simple contingency table-based methods, such as the Χ^2^ test, do not allow for covariate adjustment and rely on the assumption of independence between observations, and are therefore not appropriate methods to analyse CRTs [31–34]. Regression-based methods can be used to obtain covariate-adjusted effect estimates. Logistic regression is a widely used approach for analysing binary outcomes within a regression framework, which uses the canonical logit link function to obtain an estimate of a covariate-adjusted OR. In a CRT setting, extensions of logistic regression to clustered data include generalised linear mixed models (GLMM), generalised estimating equations (GEE) [35], or generalised linear models (GLMs) with cluster-robust standard errors (CRSE), and can be used to obtain valid covariate-adjusted ORs and inferences that account for the clustering.

Estimating adjusted RRs or RDs is less straightforward than estimating ORs with logistic regression and models often have poor convergence, posing a challenge for statisticians who wish to present these measures. In CRTs, RRs and RDs can be estimated with generalised regressions with a log or identity link respectively, by using cluster-level summaries, or by using post-estimation transformation of logistic regression coefficients. Since the CONSORT guidelines advocate reporting absolute measures for all binary outcomes, it is essential for trialists to consider appropriate methods for estimating RDs. Reviews of published RCTs and CRTs indicate that the OR is the most reported adjusted relative effect measure [16, 23]. However, where unadjusted relative effects are reported, these are more often RRs [16, 23]. The relative scarcity of adjusted RRs and RDs in the literature may reflect the additional complexities involved in their estimation and the challenges of incorporating covariate adjustment within a regression framework [36].

### Methods to estimate a RR or RD in a CRT

RRs and RDs in CRTs can be estimated using either analyses based on cluster-level summaries or with analysis using individual-level data [37].

### Cluster-level methods

Cluster-level methods use a summary of the outcome at the cluster level and treat the clusters as the independent units for comparison. This approach typically involves methods such as a t-test, or linear regression to allow for adjustment with cluster-level covariates. A degree of freedom correction is usually needed to account for the small number of clusters and additional cluster-level covariates. Cluster-level methods are necessary when only aggregate data are available (such as cluster specific prevalence) but can also be used when individual-level data are available. Individual-level covariates can be adjusted for in a cluster-level analysis by using a two-stage approach. First, a logistic regression adjusting for the individual level covariates ignoring the treatment indicator is fitted to obtain each individual’s predicted probability of the outcome, then a ratio-residual or difference-residual for each cluster is estimated by contrasting the expected number of events with the observed number of events [37–39]. These ratio-residuals or difference-residuals can be then compared using typical cluster-level methods such as a t-test to estimate an individual covariate-adjusted RR or RD. Cluster-level methods typically target a cluster-average estimand, which gives equal emphasis to each cluster, but can be modified to target a participant-average estimand by weighting by cluster size or by using binomial regression (if denominators are available) [39]. Cluster-level methods are a good choice when the number of clusters is small, as they often provide better control of type I error rates compared to individual-level methods [40].

### Binomial regression

Binomial regression is a method of estimating RRs or RDs using a GLM framework using a binomial distribution. Unlike logistic regression, which uses the canonical logit link and estimates ORs, a log link or identity link function can be used to estimate RRs or RDs respectively [41], and can be implemented with GLMMs, GEEs or with CRSEs to account for the correlations due to clustering. Log-binomial and identity-binomial models are straightforward to understand and produce valid model-based standard errors, but they often suffer from poor convergence, and can predict individual probabilities greater than one [41–45].

### Modified poisson regression

An alternative to binomial regression for estimating RRs is the modified Poisson model [46, 47]. In this method, a Poisson regression is fitted to the Poisson distribution using the canonical log link, and, as for binomial regression, a GLMM, GEE, or GLM with CRSE can be used to account for clustering. The model-based standard errors rely on the Poisson distribution where the variance equals the mean. However, the standard Poisson model assumes that the variance equals the mean which is violated when the data are truly binomial, especially when the outcome is common, leading to overestimation of the standard errors [46]. This misspecification can be corrected using a heteroscedastic consistent (HC) robust ‘sandwich’ estimator, hence the name ‘modified’ Poisson [48]. The modified Poisson method often exhibits better convergence than the log-binomial models while still providing consistent estimates of the RR and valid standard errors under mild regularity conditions [42, 46, 49].

### Substitution

Substitution is a two-stage method of estimating a RR after fitting a logistic regression, using a transformation equation involving the OR and the prevalence of the outcome in the control arm [50]. This method is straightforward and correctly estimates the point estimate for a RR but fails to consider the variability of the prevalence in the control arm when estimating the standard errors. Reviews for individual RCTs have demonstrated that this method consistently produces biased estimates of the standard errors, and should therefore not be considered as a method for use in trials [44, 51–53]. Reviews of methods for use in CRTs usually do not consider this method due to the known biases in the standard error estimation.

### Marginal standardisation

An alternative approach to obtain RRs and RDs in RCTs from logistic regression is marginal standardisation, or ‘G-computation’ [44, 54]. Here, predicted probabilities of the outcome are made from a logistic GLMM or GEE for every participant under both the treatment and control conditions, which are then averaged and contrasted to estimate a marginal population-averaged RR or RD. Confidence intervals are typically estimated using the delta method, although with rare outcomes (under 10%) and small samples (n < 100) this suffers from under-coverage. An alternative is bootstrapping, which shows appropriate coverage in simulation studies in an RCT setting [44, 54], or using ‘unconditional’ standard errors if fitting random effects models [55]. Marginal standardisation has been used in recent CRTs, and has been shown to robustly target an individual-average or cluster-average estimand in a CRT setting [1, 56].

### Small sample corrections

In CRTs, it is common to have a limited number of clusters, with reviews suggesting the median number of clusters could be as low as 12 [6, 40, 57–59]. GLMMs and GEEs are based on asymptotic theory, assuming a large number of clusters. When the number of clusters is small, these models are at risk of inflated type 1 errors and *p*-values that are too small; thus, corrections should be made to improve their performance. Generally, if there are fewer than 30–40 clusters for GLMMs or 40–50 clusters for GEE, small-sample corrections are recommended [6, 57, 60, 61]. Small-sample corrections typically modify either the standard errors or the degrees of freedom for t-distribution based inference, and many different methods have been proposed, such as Between-Within, Kenward-Roger or Satterthwaite for mixed models, or Fay-Graubard, Kauermann-Carrol, or Mancl-deRouen for GEE [6, 40, 62–65]. The performance of each correction depends on the several factors, including outcome prevalence, variation in cluster size, variation in outcome prevalence across cluster, and the intraclass correlation (ICC), and most reviews evaluated small-sample corrections when estimating ORs, meaning there is little empirical evidence supporting specific recommendations for RRs or RDs [66].

### Aims and objectives

Despite the clear importance of reporting RRs and RDs in CRTs, these measures are reported less frequently than ORs and are rarely presented as covariate-adjusted estimates. To adhere to guidelines set out by CONSORT and to uphold good reporting practice, trialists should express their effect estimates in both relative and absolute terms. However, recent reviews find that the standard of reporting remains poor, and only one review to date has explored which methods are used to estimate RRs and RDs in RCTs [36]; no such review has looked specifically at CRTs. Estimating RRs and RDs is generally more challenging than ORs and are further complicated in a CRT setting due to additional complexities such as clustering, small sample considerations, and convergence issues in regression models.

This protocol outlines the methods for a review of the statistical methods that are used to estimate RRs and RDs in published CRTs. We will review published CRT reports with the following objectives:

### Primary objective

1. Identify and describe in detail the statistical methods used in published CRT reports to estimate unadjusted and adjusted RRs and RDs, and to estimate the proportion of trials that use each method, including the methods to estimate confidence intervals, standard errors and *p*-values.

### Secondary objectives

2. Estimate the proportion of published CRTs with binary outcomes that report RRs or RDs.
3. Estimate the proportion of published CRTs with binary outcomes that report both an absolute and relative effect measure, thereby directly assessing adherence to this item of the CONSORT guidance.
4. Assess whether clustering is accounted for in the analysis of RRs and RDs by examining the use of random effects, GEEs, CRSEs, or alternatives.
5. Determine how frequently, and which type of, small-sample corrections are used in CRTs that report a RR or RD.
6. Assess how covariates are handled in CRTs reporting RRs or RDs by examining whether trials adjust for covariates and how many covariates are included.

## Methods

We will conduct a review of published trial reports of CRTs. A previous comprehensive review conducted by Goldstein and colleagues identified 800 CRTs that were conducted in low- and middle-income countries and published between 2017 and 2022 in all journals [67]. For efficiency and convenience, to avoid the need for a new full literature search, we will use this database of trials as the basis for our review.

### Inclusion criteria

The full inclusion criteria of the original systematic review can be found in the original open-access paper [67]. Briefly, the authors searched MEDLINE for CRTs evaluating health-related interventions, conducted exclusively in low- and middle-income countries (LMICs), and published in English between 2017 and 2022. From this database, we define two samples of articles: a broad binary outcome sample for objective 2, and a nested RR/RD sample (which is a subset of the binary outcome sample) to answer all other objectives.

For the binary outcome sample, we will select articles for inclusion to this review if they meet the following criteria:

- A CRT with a parallel, or parallel with baseline, design
- Have a primary outcome which is binary

We will define the nested RR/RD sample as a subset of the binary outcome sample that also meets the following criterion:

- Reports a RR or RD for the primary outcome

We have limited this review to parallel designs only, as this review is focusing on the analytical methods used to estimate RR and RD. More complex multi-period designs, such as stepped-wedge trials, may use similar underlying models; however, their designs include additional complexities which should be accounted for in the analysis methods such as time confounding and between-period correlations. As these complexities are not the primary focus of this study, they have been excluded to ensure a clear and focused review. Of the 800 trials in the original review, 716 (90%) were parallel or parallel with baseline.

This review is limited to trials that have a primary outcome which is binary. We include a broad definition of ‘primary outcome’ because we anticipate that many reports will not clearly define a single primary outcome (especially because the original review included trials published in all journals, including ones that do not have a mandatory requirement to adhere to CONSORT guidance). To this end we have allowed some flexibility to include trials where a binary outcome appears to be the focus without this being stated explicitly. We will determine which outcome is the primary outcome using the following hierarchy:

1. An outcome explicitly stated to be the primary (or a joint/dual primary) outcome;
2. The first outcome used for the sample size calculation;
3. The first outcome listed as an ‘outcome’ or ‘endpoint’ in the methods section;
4. The first outcome listed in a table of results where comparisons between trials arms are made

If the same binary outcome is measured at multiple timepoints and no primary timepoint is specified, the first post-baseline timepoint will be selected. For the purposes of this review, we will hereinafter refer to the outcome which has been chosen using this hierarchy as the primary outcome, even if it is not explicitly stated as such in the text.

Trials in which a primary binary outcome cannot be determined using this hierarchy will be excluded, as we expect the statistical methods for these more exploratory outcomes to be less thoroughly described than for primary outcomes.

The outcome will be considered binary if it is inherently binary at the individual level, irrespective of whether a cluster-level summary measure was collected and analysed. For example, outcomes which are collected and reported as ‘proportion of the cluster experiencing an event’ will be considered binary. We will not include outcomes which can be expressed as counts per patient (e.g. “rate of hospital visits over 12 months”), unless they are dichotomised at the patient level (e.g. “proportion of patients visiting hospital at least once in 12 months”).

Only trials in the RR/RD sample will undergo full data extraction. Defining these two samples allows us to fully describe the screening process and provide a clear denominator when reporting the proportion of trials using different statistical methods to estimate RRs and RDs.

### Sample size

**Fig. 1:**
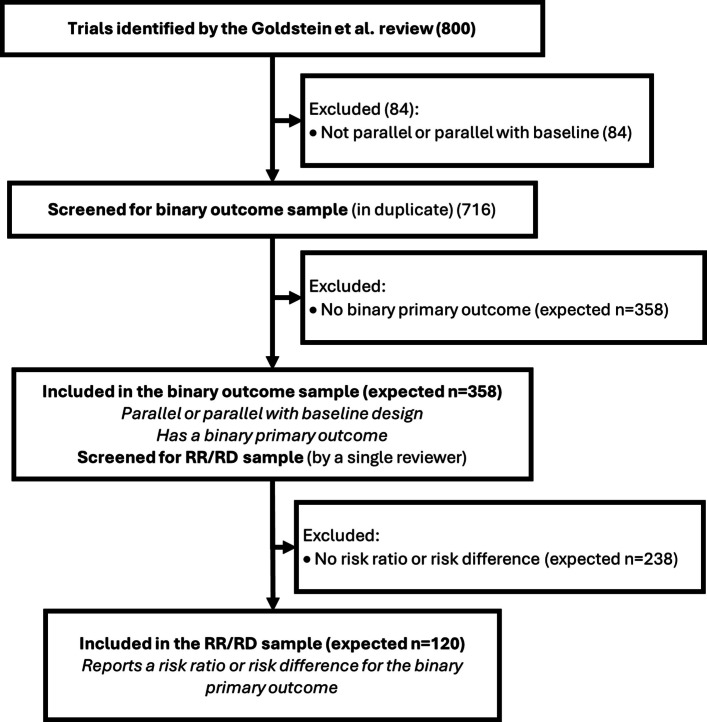
Flowchart of screening and included samples of trials

Of the 800 studies included in the review by Goldstein et al., 716 (90%) are parallel or parallel with baseline. Of these, we anticipate that approximately half (~ n = 716/2 = 358) will include a primary outcome which is binary (the binary outcome sample), based on previous reviews of RCTs and CRTs which show similar rates [5, 6]. Of the trials with a binary outcome, we expect around one third to each present a RR or a RD (the RR/RD sample) [16] (Fig. 1). This gives us an approximate expected total sample size of 120 trials which will be included in the RR/RD sample, although the exact number will depend on the specific sample of trials in the original review. Using the normal approximation for the variance of proportions and assuming proportions of 50% (to give the most conservative estimate of precision), 120 trials will allow us to estimate the percentage of studies employing each statistical method with reasonable precision (95% confidence intervals narrower than plus or minus 10 percentage points from the point estimate of the mean).

### Screening and data extraction

Data for the original systematic review was collected and stored in Airtable (www.airtable.com), a cloud-based collaborative database platform. We have obtained permission from the original authors to access and use this data for our study, and we will continue to use the same platform for screening and data extraction.

Screening and extraction will occur in two stages. The first stage will be conducted in duplicate and will be used to identify trials that meet the criteria for inclusion in the binary outcome sample. For the first stage (identification of the binary outcome sample), the dataset will be filtered to include only the 716 trials with a parallel design. Each trial will then be assigned a random floating-point number between 0 and 1, which will be used to sort the trials in a random order. Screening batches of size 30 will be sequentially assigned in ascending order based on this random number and allocated to two screeners based on their availability. Authors JAH, JFS, YW, and MT, plus two additional collaborators (named in acknowledgements) will be involved in screening. Screening will be conducted in duplicate, using a screening proforma in Airtable. After completing each batch, the two screeners will meet to resolve any discrepancies, and any remaining disagreements will be referred to a third screener to mediate a final resolution.

The second stage will involve identifying which effect estimates are reported for all trials with a binary outcome, then for those reporting a RR or RD extracting details on trial characteristics and the estimators. Extraction will follow a structured data extraction proforma in Airtable, which is included in the supplementary material (Appendix A), with the data items outlined in the next section. Data will be extracted from the published primary trial reports and if not clear from the primary trial report, protocols and statistical analysis plans (SAPs) if available. Extraction will be conducted in batches in a random order, assigned using the same method as for screening but using a different, unrelated random number.

The second stage of extraction will begin with an internal pilot phase, where each reviewer involved in the data extraction will independently extract the same small batches of two to five trials in parallel. After each pilot batch, all reviewers will meet to discuss any discrepancies and refine the wording of the data extraction proforma accordingly. The pilot will continue iteratively until there is a consensus amongst the reviewers that the proforma’s wording enables consistent and reliable extraction. The pilot extractors will include JAH, JFS, YW, MT, KH, SW, plus additional collaborators (named in the acknowledgements).

After the pilot, full data extraction of the remaining trials will be conducted by a single reviewer for each trial, for resource and feasibility reasons. The full set of included trials will be divided among the reviewers based on their availability and capacity, with each trial extracted only once without duplication. At least 10% of the extractions will be reviewed by one author (JAH) throughout the extraction period; the study leads may recommend further training of extractors or duplicate extraction for the full sample if consistency is poor.

### Data items

For trials reporting a RR or RD, the data items extracted will consist of trial characteristics, the prevalence and magnitude of the effect for the chosen primary outcome, and the details of the statistical methods used in the estimation of the RR or RD. If trials report both an adjusted and unadjusted estimate for the same outcome, we will focus the extraction on the adjusted estimate.

### Trial characteristics

The trial characteristics extracted for this study will include:

- Number of trial arms
- Number of clusters randomised and number of clusters used in the analysis
- Total sample size (the number of participants assessed for the primary outcome).
- Number of covariates involved in the randomisation

Additionally, the original Goldstein et al. review extracted trial characteristics which will also be summarised to provide further descriptive context:

- Year of publication
- Unit of randomisation (geographical area, primary care clinics/settings, schools/classrooms, hospitals or specialist care settings, professionals, workplaces, childcare institutions, residential complexes, mixed units of randomisation, other)
- WHO region of trial conduct (Africa, Sout-East Asia, Western Pacific, Americas, Eastern Mediterranean, Europe, Multiregional)

### Primary outcome

Further details about the specified primary outcome will include:

- The unadjusted prevalence of the outcome at the primary follow-up timepoint, by arm if provided, aggregated otherwise

### Statistical methods used to estimate RRs and RDs

For each effect estimate, we will extract the statistical method used:

- For cluster-level methods
  - ◦ Method used for the analysis (t-test on cluster-level summaries, t-test on ratio or difference residuals, other)
- For regression-based methods
  - ◦ The distribution and link function (log-binomial, identity-binomial, log-Poisson, identity-Poisson, identity-Gaussian, other)
  - ◦ Method used to address clustering (GLMM, GEE, GLM with CRSE, GLM with fixed effect for cluster, not accounted for)
- For GLMMs
  - ◦ The method used to estimate the standard errors (model-based, robust ‘sandwich’, Kenward-Roger, other)
  - ◦ The method used to estimate degrees of freedom (no correction, Kenward-Roger, Satterthwaite, Between-Within, other)
- For GEEs
  - ◦ The working correlation structure (exchangeable, independent, unspecified, other)
  - ◦ The method used to estimate the standard errors (robust ‘sandwich’ estimator, Kauermann-Carrol, Fay-Graubard, other)
  - ◦ The method used to estimate degrees of freedom (no correction, Between-Within, Fay-Graubard, other)
- For all methods
  - ◦ The number of covariates used in the analysis, and, if restricted randomisation was used, whether all restricted randomisation covariates were used in the analysis
  - ◦ Whether the analysis adjusted for a baseline measure of the primary outcome
  - ◦ Whether any, and which, weighting was used
  - ◦ Whether the authors state that convergence forced a change from the pre-specified methods
  - ◦ Software package, and the code if it is available

For all extracted data items, an option for ‘unclear/not reported’ will be provided to allow for cases where there is genuine uncertainty, which we have not reported in the above group summaries for brevity. If both reviewers select ‘unclear’, we consider this agreement for the purpose of consensus monitoring.

### Analyses

Categorical variables will be summarised with frequencies and percentages. Continuous variables will be summarised with medians and interquartile ranges (IQR). For the primary objective, percentages will be estimated with 95% confidence intervals using the exact Clopper-Pearson method [68]. Confidence intervals will not be estimated for any other objectives. A separate template report (Appendix B) will outline the format of the tables and figures used to present the results of this study.

## Discussion

### Recap of study aims

Binary outcomes are common in CRTs, and the CONSORT guidelines recommend that authors report both an absolute and relative effect measure for binary outcomes. For relative measures, authors generally choose between ORs or RRs, but absolute effect measures are typically RDs (or NNT, which is derived from the RD and is not estimated directly). Adherence to these guidelines is generally poor and, when reported, RRs and RDs are often not adjusted for covariates. ORs can be estimated directly via logistic regression, but the methods required to estimate RRs and RDs are more complex and less widely understood. RRs and RDs require alternative modelling approaches, often with additional considerations around convergence, covariate adjustment, and accounting for clustering. This study aims to capture current practice around the estimation of RRs and RDs in published CRTs.

### Anticipated outcomes

This study is similar in its intended output to a 2025 review by Thompson et al., which examined the methods used to estimate RRs and RDs in individually randomised trials, except this study is specific to CRTs [36]. The Thompson review found that among trials reporting a RR or RD, only half (150/308) presented an effect estimate that was adjusted for covariates. Reporting of statistical methods was poor: 79% of adjusted RRs and 61% of adjusted RDs clearly described the method used, while only 32% and 28% of unadjusted RRs and RDs respectively were reported with clear methods. When described, the most common approach was binomial regression, with a log link for RR and identity link for RD. Modified Poisson was used in 29% of trials reporting an adjusted RR and 23% of trials reporting unadjusted RRs, although this approach was much less commonly used to estimate RDs. Marginal standardisation and linear models were occasionally used to estimate RDs (7% and 11% respectively) but were rarely used to estimate RRs (1 trial and 0 trials of 30 respectively).

We anticipate that the methods used in CRTs will be broadly similar to those observed by Thompson et al. when individual level regression based approaches are used. Where regression-based methods are used, we expect many may not appropriately account for clustering (via the use of random effects, GEEs, or cluster robust standard errors). The Thompson review found that many individually randomised trials do not adjust for covariates that are prognostic, associated with missing data, or used in restricted randomisation, and we expect the same in CRTs particularly as regression methods are more complex due to clustering. If this were the case, the trials results may be at risk of incorrect type 1 errors, lost power, or bias [4, 25, 27–34, 69–75]. Cluster level methods are an additional class of methods that would not be considered in individual RCTs, and these may be quite commonly used as their implementation is straightforward in standard statistical software.

### Strengths and limitations

This study uses a previously collected dataset of CRTs. This was a pragmatic decision based on resources and time constraints and makes the review feasible. The Goldstein review has several methodological strengths that also carry over to this study: it was a comprehensive systematic review with duplicate screening, high interrater reliability, and inclusion of all journals indexed in MEDLINE. This broad scope enhances generalisability beyond high-impact journals, reflecting a wider range of trial quality and reporting practices. The authors of the original review explicitly noted that their dataset could serve as a foundation for future methodological studies, such as the one outlined in this protocol.

There are however limitations to relying on this specific sample. First, the review specifically looked at trials conducted in LMICs. This limits the generalisability of the findings, as trials conducted in high-income countries (HICs) may have different access to statistical expertise, infrastructure and tools, although we note that many trials recruiting in LMICs have collaborators based in HICs. The sample is restricted to trials published between 2017 and 2022, which may miss more recent shifts in methodological practice or uptake of newer methods. Additionally, the original review focused exclusively on CRTs evaluating health-related interventions; cluster trials are common in other fields such as economics, public policy or marketing. Since researchers tend to remain in their own discipline, methodological practices across domains may differ substantially. As a result, the findings of this study are likely generalisable only to healthcare-related CRTs and may not reflect practices in other fields.

In addition to limitations related to the study that produced the underlying dataset, there are methodological considerations specific to the design of this review. Whilst screening is conducted in duplicate, full data extraction is carried out by a single reviewer per trial. This was a pragmatic decision based on resources and feasibility, given the quantity of the information being extracted. To mitigate the risks associated with single extraction, we have included an iterative pilot phase involving multiple reviewers (including additional collaborators who will not be reviewers in the main extraction to provide external insight). This should ensure there is consistency in the interpretation and provide clear guidance and instructions for the reviewers.

We have restricted the review to CRTs with parallel (or parallel with baseline) designs. Although more complex designs such as stepped wedge are increasingly common, they form a small proportion of the original sample. These designs introduce additional complexities which may dominate the statistical methods section and make it difficult to isolate the underlying method for estimating RRs or RDs.

We have limited this review to trials with a binary primary outcome, rather than including any trial that reports any binary outcome, as we expect that statistical methods are most likely to be clearly described for a primary outcome. We recognise that many trials do not clearly define a single primary outcome, or list multiple outcomes as primary. To address this, we have developed a structured hierarchy to identify the outcome that appears to be the main focus of the analysis or interpretation. This approach is intended to maximise the chance that the analysis methods are properly described in the trial manuscript. Even after isolating the most relevant outcome, reporting of statistical methods may still be limited due to journal word limits or poor reporting. To mitigate this, we will also review protocols and SAPs, where available, to supplement and clarify the information presented in the main trial report.

We will collect information on the software packages and versions used, as well as any statistical code if it is available, but we expect access to underlying code to be limited. As a result, it will not be possible to verify whether the analysis was implemented in a way that accurately reflects the methods described. This limitation is particularly relevant when estimating RRs and RDs in CRTs, as many of the approaches, such as models with non-canonical link functions, robust standard errors, or random effects, are not straightforward to implement and are often not supported by default procedures. Furthermore, implementation varies across commonly used statistical software. For example, Stata allows robust standard errors in GLMMs with relative ease, while R requires additional packages or custom functions, and SAS syntax can vary depending on the procedure used. Without access to code or detailed documentation, it is not possible to determine whether these complexities have been addressed correctly in practice.

In addition to the limitations around implementation, we will not be collecting information on the target estimands of each trial. Estimands in CRTs are more complex than in individually randomised trials, due to distinctions such as participant-average versus cluster-average effects, and marginal versus conditional targets, both with respect to covariates and to the cluster itself. Different statistical methods can target different estimands, and some can be weighted or specified to change the target of inference [39]. However, this level of distinction is rarely discussed in trial publications, and the reporting of target estimands in both RCTs and CRTs has historically been poor [39]. As a result, we are unable to systematically assess whether the estimators used in each trial are aligned with a clearly defined, intentional estimand.

### Future directions

Previous simulation studies have evaluated methods for individually randomised trials without random effects [76, 77], unadjusted estimates in CRTs [78], small-sample corrections for odds ratios in CRTs [65, 79], marginal standardisation in CRTs with no small-sample considerations [80]. However, there are gaps in the empirical evidence supporting the use of different methods, particularly around small sample considerations, integration and likelihood methods, and differences between software implementations. The descriptive findings from this review can serve as a justification for further simulation studies to evaluate the performance of both commonly used and underused methods. These simulations could assess bias, coverage and convergence under realistic CRT scenarios, including small numbers of clusters, high ICC, or rare outcomes. Exploring the impact of covariate adjustment on the validity and efficiency of RR and RD estimates would further support evidence-based recommendations.

There may also be a need for practical guidance to help statisticians in selecting and implementing appropriate methods for estimating RRs and RDs in CRTs. This could include documentation of how default procedures differ across software platforms such as R, Stata, and SAS, particularly with respect to the handling of random effects or where non-canonical link functions may not be natively supported. Developing worked examples, code libraries, and decision tools would reduce misapplication of methods and support reproducibility. These resources could feed into training materials and updates to reporting guidance.

Another potential area of interest is the link between pre-specified estimands, and the analysis methods used. While this review focuses on the analysis as reported, it does not assess whether the chosen methods align with a clearly defined estimand. Estimands are increasingly recognised as key to linking the scientific question, study design, and analysis, but most of the trials in this review were designed and reported before this framework became widely adopted. It is therefore unlikely that the authors selected their analysis methods with an explicit estimand in mind, and choices were more likely driven by familiarity, software defaults, or ease of implementation. Although the distinction between marginal and conditional model targets are less important for RRs and RD than for ORs, as they are collapsible effect measures, these measures can still differ depending on whether a participant-average or cluster-average effect is targeted [81]. Certain analytical choices can imply different targets; for example, independence GEEs target an individual-average effect, whereas a cluster-average effect can be estimated with an unweighted cluster-level analysis or a weighted independence estimating equation [39]. Other approaches such as GLMMs or GEEs with exchangeable correlation structures are data dependent and do not target a clear estimand under empirical informative cluster size [39]. Although we extract enough aspects of an estimator to describe features related to its target, we will not attempt to infer or classify estimands for each trial, as doing so would risk over-interpretation given the limited reporting and the likelihood that these choices were not intentional. Future work could involve reviewing protocols and SAPs to evaluate whether estimands are clearly defined and consistently carried through to the analysis.

Finally, while this review is limited to CRTs conducted in LMICs and in the healthcare domain, including trials conducted in HICs or other disciplines could identify methods which are less commonly used in healthcare and provide empirical support for further investigation.

## Conclusion

This review will provide a comprehensive overview of how RRs and RDs are estimated and reported in CRTs with binary outcomes, highlighting current practice in relation to covariate adjustment, clustering, and small-sample considerations. The findings will inform future methodological research, support the development of practical guidance, and contribute to improved analysis and reporting standards in CRTs.

## Data Availability

Data generated and analysed during this review will be made available by the corresponding author on reasonable request. Access may require permission from the authors of the original database used as the basis for this study.

## Abbreviations

CI: Confidence Interval
CONSORT: Consolidated Standards of Reporting Trials
CRSE: Cluster-Robust Standard Error
CRT: Cluster Randomised Trial
GEE: Generalised Estimating Equation
GLM: Generalised Linear Model
GLMM: Generalised Linear Mixed Model
HC: Heteroscedasticity-Consistent
HIC: High-Income Country
ICC: Intraclass Correlation Coefficient
IQR: Interquartile Range
LMIC: Low- and Middle-Income Country
NNT: Number Needed to Treat
OR: Odds Ratio
RD: Risk Difference
RCT: Randomised Controlled Trial
RR: Risk Ratio
SAP: Statistical Analysis Plan
SD: Standard Deviation
SE: Standard Error
WHO: World Health Organization
